# Ultrasound findings and clinical evaluation of temporomandibular joint involvement in systemic lupus erythematosus and Sjögren’s syndrome: A cross-sectional study

**DOI:** 10.1097/MD.0000000000049215

**Published:** 2026-06-12

**Authors:** Somayeh Soroureddin, Ahmadreza Jamshidi, Omid Ghaemi, Tahereh Faezi, Ali Baradaran Bagheri, Majid Alikhani

**Affiliations:** a Department of Internal medicine, Emam Ali Hospital, Alborz University of Medical Sciences, Karaj, Iran; b Rheumatology Research Center, Shariati Hospital, Tehran University of Medical Sciences, Tehran, Iran; c Department of Radiology and interventional radiology, Imam Khomeini hospital, Tehran University of Medical Sciences, Tehran, Iran; d Department of Radiology and interventional radiology, Shariati hospital, Tehran University of Medical Sciences, Tehran, Iran; e Department of Neurosurgery, Shahid Madani Hospital, Alborz University of Medical Sciences, Karaj, Iran.

**Keywords:** connective tissue disorder, Sjögren’s syndrome, systemic lupus erythematosus, temporomandibular joint, TMJ dysfunction, ultrasound

## Abstract

This study aimed to evaluate temporomandibular joint (TMJ) involvement in patients with systemic lupus erythematosus (SLE) and Sjögren’s syndrome, using clinical assessment and ultrasound (US) imaging. A cross-sectional study was conducted involving 51 patients (41 with SLE and 10 with Sjögren’s syndrome). TMJ dysfunction was assessed using clinical examination and US. Statistical analyses compared clinical and ultrasound findings and explored associations with disease characteristics. TMJ abnormalities were detected in 57% by US and 39% by clinical examination. Cortical irregularities (46% in SLE, 60% in Sjögren’s syndrome) and osteophyte formation (29% in SLE and 30% in Sjögren’s syndrome) were the most common US findings. Joint effusion was observed in 9.8% of patients with SLE but was absent in those with Sjögren’s syndrome. Clinical symptoms, such as TMJ pain (7.3%), tenderness (2.4%), and joint clicking (4.9%), were reported only in patients with SLE. Jaw deviation was the most frequent clinical finding (26.8% in SLE and 30% in Sjögren’s syndrome). TMJ abnormalities were more frequently detected via US than by clinical examination, particularly in Sjögren’s syndrome (70% vs 30%, *P* < .05). TMJ involvement is prevalent in both SLE and Sjögren’s syndrome, with US detecting abnormalities at a higher rate than clinical examinations. These findings highlight the importance of US in early TMJ assessment, supporting its role as a valuable screening tool for rheumatic disease management. Further research is required to explore the clinical implications and disease-specific patterns of TMJ involvement.

## 1. Introduction

The temporomandibular joint (TMJ) is a complex ginglymoarthrodial articulation essential for mastication, speech, and other orofacial functions.^[1]^ Owing to its intricate anatomy, the TMJ is prone to a variety of disorders, including internal derangements, that can lead to pain, dysfunction, and, if undiagnosed, irreversible joint damage. Temporomandibular disorders affect approximately 5% to 12% of the United States population and have been reported in up to 56% of Iranian adults in prior studies.^[2,3]^ These encompass a spectrum of conditions ranging from myogenous dysfunction to articular disc derangements and osteoarthritic changes.^[4]^

As a synovial bicondylar joint, the TMJ may be secondarily affected by inflammatory arthropathies and systemic rheumatic diseases, a clinical problem that is increasingly recognized yet remains underappreciated.^[5]^ Since the early stages of TMJ arthritis may be asymptomatic and elude detection during routine examinations, imaging modalities are pivotal for early identification and management of joint pathology. Moreover, the systemic, multi-organ nature of rheumatic diseases, such as systemic lupus erythematosus (SLE) and Sjögren’s syndrome, often results in less clinical focus on TMJ involvement, contributing to a lack of comprehensive treatment plan for affected patients.

Conventional imaging techniques, such as plain radiography and computed tomography (CT), primarily reveal advanced osseous changes and lack sensitivity for detecting soft tissue alterations and early inflammatory signs. Magnetic resonance imaging (MRI) is widely regarded as the “gold standard” for TMJ assessment because it can identify both acute inflammatory changes (e.g., synovitis, joint effusion, bone marrow edema) and chronic degenerative alterations (e.g., condylar erosion and disc abnormalities).^[3]^ However, MRI is limited by cost, longer acquisition times, and patient discomfort (e.g., TMJ pain during positioning or claustrophobia). Ultrasound (US) has recently emerged as a promising alternative for TMJ evaluation. It offers several advantages: it is cost-effective, widely available, free of ionizing radiation, and enables dynamic, real-time assessment of the TMJ. The relatively brief examination time (10–15 minutes) and the ability to perform dynamic assessments make US particularly attractive for routine screening. US may be particularly useful for early or subclinical TMJ involvement in rheumatic diseases, where imaging abnormalities can precede overt symptoms.

The literature contains several reports on TMJ involvement in certain rheumatic diseases, mainly RA, psoriatic arthritis, and ankylosing spondylitis, along with several studies on various aspects of the condition.^[6–8]^ However, less attention has been given to other rheumatic diseases, especially SLE and Sjögren’s syndrome, particularly with US is used as the imaging modality, for which disease-specific studies remain limited. The goal of this study was to elucidate the severity of TMJ involvement in SLE and Sjögren’s syndrome by exploring associations between disease characteristics and TMJ dysfunction measured clinically and by US, and by examining concordance between US findings and clinical TMJ involvement.

## 2. Methods

### 2.1. Study design

This cross-sectional study was conducted to evaluate TMJ involvement in patients diagnosed with SLE or Sjögren’s syndrome. The study was carried out at the Rheumatology Clinic of Shariati Hospital over an eleven-month period, from April 2023 to February 2024. This research focused on assessing TMJ dysfunction through both clinical examination and US imaging to determine the severity and frequency of TMJ involvement in these 2 rheumatic diseases.

### 2.2. Study population

The study population consisted of patients with confirmed diagnoses of SLE and Sjögren’s syndrome who were referred to the Rheumatology Clinic at Shariati Hospital. SLE was diagnosed based on the Systemic Lupus International Collaborating Clinics classification criteria, while Sjögren’s syndrome was diagnosed according to the American College of Rheumatology/European League Against Rheumatism (ACR/EULAR) classification criteria. Although this resulted in an unequal distribution between groups (41 SLE vs 10 Sjögren’s syndrome), this imbalance reflects the stricter exclusion criteria applied to the Sjögren’s syndrome cohort, particularly the exclusion of secondary Sjögren’s syndrome associated with rheumatoid arthritis, which is common in our referral setting.

To ensure a homogeneous study population and minimize confounding factors, a total enumeration approach was used to include all patients who met the inclusion criteria. Patients were excluded if they had a history of jaw trauma, withdrew written informed consent to be included in the study, previous jaw surgery, recent oral or dental infections, congenital craniofacial anomalies, recent dental procedures (within the past 2 weeks), jaw abscesses, ongoing orthodontic treatment, a history of migraines, cervical spine or shoulder surgery, or a concurrent diagnosis of rheumatoid arthritis. These exclusion criteria were applied to eliminate any conditions that could independently contribute to TMJ dysfunction and potentially interfere with the study findings. Written informed consent was obtained from each participant prior to inclusion. This study was approved by the Ethics Committee of the Tehran University of Medical Sciences (Approval No. IR.TUMS.MEDICINE.REC.1402.47).

### 2.3. Data collection

After obtaining written informed consent from each participant, detailed demographics and clinical information were collected using a structured questionnaire. This questionnaire included variables such as age, sex, and disease duration, along with relevant clinical characteristics including major organ involvement, history of arthritis, and medication use. In addition, laboratory data, such as rheumatoid factor (RF), erythrocyte sedimentation rate (ESR), C-reactive protein (CRP), anti-cyclic citrullinated peptide (anti-CCP), complement level status (low C3, C4, or CH50 levels), and anti-double-stranded DNA (anti-dsDNA) antibodies were recorded at the time of the study. The SLE Disease Activity Index (SLEDAI) was used to monitor the intensity of the condition in patients with SLE.

A comprehensive clinical assessment of the TMJ function was performed by an experienced rheumatologist. This evaluation included an assessment of TMJ pain (focal pain at rest or upon jaw movement), joint tenderness (bilateral palpation of the temporalis and masseter muscles and the lateral pole of the condyle), the presence of clicking sounds during mandibular movement, jaw deviation upon opening (defined as a visible lateral shift of the mandible >2 mm from the midline during opening). Trismus was defined as maximum interincisal opening below approximately 35 mm.^[9,10]^ The clinical findings were systematically documented for further comparison with the US results. Data completeness for physical examinations and US measurements was verified at the time of enrollment and none had missing values, and no imputation strategies were required.

Following the clinical examination, patients underwent US imaging of the TMJ. All examinations were performed using a Samsung Medison WS80A, US system equipped with a high-frequency linear array transducer (7–12 MHz), which is standard for superficial musculoskeletal imaging in our center. Patients were positioned supine with the head turned 45 degrees to the contralateral side to maximize acoustic window accessibility. Both TMJs were scanned in the closed and open mouth positions in axial and coronal planes. To ensure consistency, joint effusion was defined as a hypoechoic area within the joint space widening the capsule-to-bone distance to > 2.0 mm. Cortical irregularity was defined as the loss of the normal continuous, smooth hyperechoic line of the condylar head, manifesting as erosions, flattening, or subcortical cysts. Osteophytes were defined as abnormal bony prominences or marginal bone proliferation on the condylar head. Disc Displacement: assessed dynamically; defined as the failure of the articular disc to interpose between the condyle and articular eminence during mouth opening. All US assessments were conducted by a radiologist blinded to the clinical assessment findings. In patients with Sjögren’s syndrome, parotid gland US was also performed to account for potential referred pain to the TMJ arising from parotid gland involvement, as salivary gland inflammation may contribute to orofacial pain.

### 2.4. Statistical analysis

All collected data were analyzed using the R software (version 4.2.3). Continuous variables are expressed as mean ± standard deviation (SD), while categorical variables are summarized as frequencies and percentages. The Shapiro–Wilk test was used to determine the normality of the continuous data distributions. Depending on the normality of the data, comparisons between groups were performed using independent t-tests for normally distributed variables or Mann–Whitney U tests for non-normally distributed variables. Categorical variables were analyzed using the chi-square test or Fisher exact test, as appropriate.

To assess the degree of agreement between clinical findings and US-detected TMJ involvement, generalized estimating equation (GEE) with an exchangeable correlation structure was used to account for correlations within repeated measures and compare TMJ involvement detected using different diagnostic modalities. Statistical significance was set at *P* < .05. Given the modest sample size, small cell counts, and multiple subgroup comparisons, covariate-stratified summaries were treated as exploratory and descriptive. No confirmatory inference regarding treatment effects was intended, and multivariable adjustment was not performed.

## 3. Results

A total of 51 patients were included in the study, of whom 41 (80%) had SLE and 10 (20%) had Sjögren’s syndrome (Table 1). Overall, 44 patients (86%) were female, and 7 (14%) were male. The mean age of the study population was 43 ± 12 years. The mean age of patients with SLE (41.59 ± 11.7 years) was lower than that of patients with Sjögren’s syndrome (48.5 ± 10.3 years). The mean disease duration was longer in SLE (10.24 ± 8.7 years) compared to Sjögren’s syndrome (6.00 ± 3.0 years); however, neither of these differences reached statistical significance (*P* = .094). The mean SLEDAI score in patients with SLE was 2.92. Elevated CRP and ESR levels, arthritis (19%), and azathioprine use were limited to patients with SLE (Table 1). RF was partially missing for several participants but was positive in 2.4% of SLE patients and 20% of Sjögren’s syndrome patients for whom this marker was available. Additionally, anti-CCP positivity was observed in 10% of patients with Sjögren’s syndrome.

**Table 1:** Baseline characteristics and drug use among included participants.

Variables		Sjogren’s syndrome (n = 10)	Systemic Lupus Erythematosus (n = 41)	All patients	*P*-value
Sex	Female	9 (90%)	35 (85%)	44 (86%)	.999
	Male	1 (10%)	6 (15%)	7 (14%)	
Age		48.50 ± 10.3	41.59 ± 11.7	43 ± 12	.094
Disease duration		6.00 ± 3.0	10.24 ± 8.7	9 ± 8	.234
Elevated erythrocyte sedimentation rate (Yes/no)		0 (0%)	8 (19%)	8 (16%)	.180
High C-Reactive Protein level (Yes/no)		0 (0%)	5 (12%)	5 (10%)	.569
Abnormally low Complement component serum levels (Yes/no)		2 (20%)	15 (37%)	17 (33.3%)	.461
Elevated rheumatoid factor (Yes/no)		2 (20%)	1 (2.4%)	3 (6%)	.227
Cyclic citrullinated peptide antibodies (Yes/no)		1 (10%)	0 (0%)	1 (2%)	.318
Arthritis (Yes/no)		0 (0%)	8 (19%)	8 (16%)	.329
Prednisolone (PDN) use		9 (90%)	40 (98%)	49 (96%)	.357
Hydroxychloroquine (HCQ) use		9 (90%)	38 (93%)	47 (92%)	.999
Azathioprine (AZA) use		0 (0%)	15 (37%)	15 (29%)	.024
Mycophenolate Mofetil (MMF) use		1 (10%)	11 (27%)	12 (23%)	.417
Rituximab (RTX) use		0 (0%)	4 (10%)	4 (8%)	.573
Cyclophosphamide (CYC) use		0 (0%)	2 (5%)	2 (4%)	.999
Methotrexate (MTX) use		0 (0%)	4 (10%)	4 (8%)	.573

*P*-values calculated from Pearson chi-square, Fisher exact test, independent t-test, or Mann–Whitney U test as appropriate.

Regarding medication use, 98% of patients with SLE and 90% of those with Sjögren’s syndrome were receiving corticosteroids at the time of the study. Hydroxychloroquine was prescribed to 93% of patients with SLE and 90% of those with Sjögren’s syndrome. Mycophenolate mofetil was used in 27% of patients with SLE and 10% of patients with Sjögren’s syndrome. Rituximab was prescribed in 10% of SLE patients, while cyclophosphamide and methotrexate were used in 5% and 10% of SLE patients, respectively. None of the patients with Sjögren’s syndrome received these medications.

US evaluation revealed various TMJ abnormalities in patients with SLE and Sjögren’s syndrome. Joint effusion was observed in 9.8% of patients with SLE but was absent in those with Sjögren’s syndrome. Osteophytes were detected in 29% of SLE patients and 30% of Sjögren’s syndrome patients, whereas cortical irregularities were noted in 46% and 60% of patients, respectively (Table 2). Disc involvement was identified in 9.8% of SLE patients and in 10% of Sjögren’s syndrome patients. Among all abnormalities, cortical irregularity and osteophyte formation were the most frequently observed in both diseases. Overall, US detected TMJ involvement in 57% of patients (defined as the presence of at least one abnormality), and the difference between Sjögren’s syndrome (70%) and SLE (54%) was not statistically significant (*P* = .483). However, the frequency of TMJ diagnosis using US (57% of all patients, 70% of Sjögren’s syndrome patients) was significantly higher than that detected through clinical examination (39% of all patients, 30% of Sjögren’s syndrome patients) for Sjögren’s syndrome (*P* < .05).

**Table 2:** Patterns of ultrasound and clinical findings in SLE and Sjogren’s syndrome patients.

Variables	Sjogren’s syndrome (n = 10)	Systemic Lupus Erythematosus (n = 41)	All patients	*P*-value
Ultrasound findings				
Effusion	0 (0%)	4 (9.8%)	4 (8%)	.573
Cyst	0 (0%)	0 (0%)	0 (0%)	Not available
Osteophyte	3 (30%)	12 (29%)	15 (29%)	.999
Cortical irregularity	6 (60%)	19 (46%)	25 (49%)	.499
Disc involvement	1 (10%)	4 (9.8%)	5 (9.8%)	.999
Ultrasound TMJ involvement (any of the above)	7 (70%)	22 (54%)	29 (57%)	.483
Clinical findings				
Pain	0 (0%)	3 (7.3%)	3 (5.9%)	.999
Tenderness	0 (0%)	1 (2.4%)	1 (2.0%)	.999
Cracking or clicking sound	0 (0%)	2 (4.9%)	2 (3.9%)	.999
Jaw deviation	3 (30%)	11 (26.8%)	14 (27.4%)	.999
Trismus	0 (0%)	2 (4.9%)	2 (3.9%)	.999
Clinical TMJ involvement (any of the above)	3 (30%)	17 (41%)	20 (39%)	.721

There was only a small subset of SLE patients who had TMJ disorder symptoms not corroborated by US findings (Table 2 and Table 3). Based on clinical examination and self-reported symptoms, TMJ pain was reported in 7.3% of SLE patients but was absent in those with Sjögren’s syndrome. Tenderness was observed in 2.4% of SLE patients, while no cases were noted in Sjögren’s syndrome patients. Joint cracking and clicking were detected in 4.9% of SLE patients and was not observed in the Sjögren’s syndrome group. Jaw deviation upon mouth opening was the most common clinical finding, present in 26.8% of SLE patients and 30% of Sjögren’s syndrome patients. Overall, clinical examination indicated TMJ involvement in 39% of patients (defined as the presence of at least one abnormality). The prevalence of TMJ involvement was lower in Sjögren’s syndrome patients (30%) compared to SLE patients (41%); however, this difference was not statistically significant (*P* = .721).

**Table 3:** Overlap of clinical and imaging findings.

	Patient subset		Ultrasound involvement		*P*-value
			No	Yes	
Clinical involvement	All patients	Yes	3 (5.9%)	17 (33.3%)	.016
		No	19 (37.3%)	12 (23.5%)	
	Systemic Lupus Erythematosus	Yes	3 (7.3%)	14 (34.1%)	.120
		No	16 (39.2%)	8 (19.5%)	
	Sjogren’s syndrome	Yes	0 (0.0%)	3 (30.0%)	.022
		No	3 (30.0%)	4 (40.0%)	

Baseline factors and serological markers were not associated with differences in detection rates between US and clinical examination in the overall cohort (Table 4). Distribution of study variables with regards to TMJ dysfunction detection method for SLE and Sjögren’s syndrome groups are also presented separately (Table 5 and Table 6).

**Table 4:** Distribution of and differences in baseline variables in all patients with regards to TMJ disorder diagnostic method.

Variable		Involvement type		Difference between involvement types	*P*-value of the difference†
		Ultrasound	Clinical		
Sex	Female	27 (61.4%)	19 (43.2%)	8 (18%; 95% CI 8.2–32.7%)	.929
	Male	2 (28.6%)	1 (14.3%)	1 (14%; 95% CI 0.4–57.9%)	
*P*-value*		.216	.223	-	-
Age (years)		45.69 ± 12.29	44.90 ± 13.97	0.80 (95% CI − 7.05 to 8.63)	.330
*P*-value		.053	.341	-	-
Disease duration (years)		10.41 ± 9.56	11.05 ± 10.89	-0.64 (95% CI − 6.74 to 5.46)	.998
*P*-value		.731	.877	-	-
ESR	Normal	22 (53.7%)	16 (39.0%)	6 (15%; 95% CI 5.6–29.2%)	.518
	High	6 (75.0%)	4 (50.0%)	2 (25%; 95% CI 3.2–65.1%)	
*P*-value		.438	.700	-	-
CRP	Normal	25 (56.8%)	17 (38.6%)	8 (18%; 95% CI 8.2–32.7%)	.545
	High	3 (60.0%)	3 (60.0%)	0 (0.0%; 95% CI 0.0–52.2%)	
*P*-value		.999	.387	-	-
Complement	Normal	20 (60.6%)	14 (42.4%)	6 (18%; 95% CI 7.0–35.5%)	.546
	Low	8 (47.1%)	6 (35.3%)	2 (12%; 95% CI 1.5–36.4%)	
*P*-value		.361	.626	-	-
RF	Negative	10 (52.6%)	8 (42.1%)	2 (11%; 95% CI 1.3–33.1%)	.999
	Positive	1 (33.3%)	0 (0.0%)	1 (33%; 95% CI 0.8–90.6%)	
*P*-value		.999	.273	-	-
Active arthritis	No	24 (55.8%)	18 (41.9%)	6 (14%; 95% CI 5.3–27.9%)	.390
	Yes	5 (62.5%)	2 (25.0%)	3 (38%; 95% CI 8.5–75.5%)	
*P*-value		.999	.456	-	-
Major organ	No	18 (51.4%)	13 (37.1%)	5 (14%; 95% CI 4.8–30.3%)	.512
	Yes	11 (68.8%)	7 (43.8%)	4 (25%; 95% CI 7.3–52.4%)	
*P*-value		.246	.654	-	-
PDN	No	1 (50.0%)	0 (0.0%)	1 (50%; 95% CI 1.3–98.7%)	Not available
	Yes	28 (57.1%)	20 (40.8%)	8 (16%; 95% CI 7.3–29.7%)	
*P*-value		.999	.514	-	-
HCQ	No	1 (25.0%)	1 (25.0%)	0 (0%; 95% CI 0.0–60.2%)	**.016**
	Yes	28 (59.6%)	19 (40.4%)	9 (19%; 95% CI 9.1–33.3%)	
*P*-value		.303	.999	-	-
AZA	No	24 (66.7%)	15 (41.7%)	9 (25%; 95% CI 12.1–42.2%)	.139
	Yes	5 (33.3%)	5 (33.3%)	0 (0%; 95% CI 0.0–21.8%)	
*P*-value		**.029**	.579	-	-
MMF	No	21 (53.8%)	14 (35.9%)	7 (18%; 95% CI 7.5–33.5%)	.957
	Yes	8 (66.7%)	6 (50.0%)	2 (17%; 95% CI 2.1–48.4%)	
*P*-value		.433	.502	-	-
RTX	No	27 (57.4%)	18 (38.3%)	9 (19%; 95% CI 9.1–33.3%)	.591
	Yes	2 (50.0%)	2 (50.0%)	0 (0%; 95% CI 0.0–60.2%)	
*P*-value		.999	.640	-	-
CYC	No	28 (57.1%)	19 (38.8%)	9 (18%; 95% CI 8.8–32.0%)	**.016**
	Yes	1 (50.0%)	1 (50.0%)	0 (0%; 95% CI 0.0–84.2%)	
*P*-value		.999	.999	-	-
MTX	No	26 (55.3%)	17 (36.2%)	9 (19%; 95% CI 9.1–33.3%)	**.016**
	Yes	3 (75.0%)	3 (75.0%)	0 (0%; 95% CI 0.0–60.2%)	
*P*-value		.625	.287	-	-

* Row-specific *P*-values calculated from Pearson chi-square, Fisher exact test, independent t-test, or Mann–Whitney U test as appropriate. Percentages and means calculated per total participants within each row; 95% Cis for categorical variables and continuous variables are exact binomial (Clopper–Pearson) and Welch t-based CI, respectively.

† *P*-value derived from GEE models fit for identifying covariate interaction with diagnostic method.

ESR = erythrocyte sedimentation rate, CRP = C-reactive protein, dsDNA = anti-double-stranded DNA, RF = rheumatoid factor, CCP = anti-cyclic citrullinated peptide, PDN = prednisolone, HCQ = hydroxychloroquine, AZA = azathioprine, MMF = Mycophenolate Mofetil, RTX: Rituximab, CYC = cyclophosphamide, MTX = methotrexate.

**Table 5:** Patient characteristics in SLE subgroup with respect to diagnostic method of TMJ disorder.

Variable		Involvement type		Difference between involvement types
		Ultrasound	Clinical	
Sex	Female	21 (60.0%)	16 (45.7%)	5 (14%; 95% CI 4.8–30.3%)
	Male	1 (16.7%)	1 (16.7%)	0 (0%; 95% CI 0.0–45.9%)
Age (years)		45.36 ± 13.04	45.94 ± 14.95	−0.58 (95% CI − 9.89 to 8.73)
Disease duration (years)		12.00 ± 10.37	12.35 ± 11.35	−0.35 (95% CI − 7.53 to 6.83)
SLEDAI		2.91 ± 2.91	2.94 ± 3.31	−0.03 (95% CI − 2.10 to 2.04)
ESR	Normal	15 (48.4%)	13 (41.9%)	2 (6%; 95% CI 0.8–21.4%)
	High	6 (75.0%)	4 (50.0%)	2 (25%; 95% CI 3.2–65.1%)
CRP	Normal	18 (52.9%)	14 (41.2%)	4 (12%; 95% CI 3.3–27.5%)
	High	3 (60.0%)	3 (60.0%)	0 (0%; 95% CI 0.0–52.2%)
dsDNA	Negative	13 (50.0%)	10 (38.5%)	3 (12%; 95% CI 2.4–30.2%)
	Positive	8 (57.1%)	7 (50.0%)	1 (7%; 95% CI 0.2–33.9%)
Complement	Normal	14 (56.0%)	11 (44.0%)	3 (12%; 95% CI 2.5–31.2%)
	Low	7 (46.7%)	6 (40.0%)	1 (7%; 95% CI 0.2–31.9%)
RF	Negative	7 (50.0%)	7 (50.0%)	0 (0%; 95% CI 0.0–23.2%)
	Positive	0 (0.0%)	0 (0.0%)	0 (0%)
Active arthritis	No	17 (51.5%)	15 (45.5%)	2 (6%; 95% CI 0.7–20.2%)
	Yes	5 (62.5%)	2 (25.0%)	3 (38%; 95% CI 8.5–75.5%)
Major organ	No	11 (42.3%)	10 (38.5%)	1 (4%; 95% CI 0.1–19.6%)
	Yes	11 (73.3%)	7 (46.7%)	4 (27%; 95% CI 7.8–55.1%)
PDN	No	1 (100.0%)	0 (0.0%)	1 (100%; 95% CI 2.5–100.0%)
	Yes	21 (52.5%)	17 (42.5%)	4 (10%; 95% CI 2.8–23.7%)
HCQ	No	1 (33.3%)	1 (33.3%)	0 (0%; 95% CI 0.0–70.8%)
	Yes	21 (55.3%)	16 (42.1%)	5 (13%; 95% CI 4.4–28.1%)
AZA	No	17 (65.4%)	12 (46.2%)	5 (19%; 95% CI 6.6–39.4%)
	Yes	5 (33.3%)	5 (33.3%)	0 (0%; 95% CI 0.0–21.8%)
MMF	No	14 (46.7%)	11 (36.7%)	3 (10%; 95% CI 2.1–26.5%)
	Yes	8 (72.7%)	6 (54.5%)	2 (18%; 95% CI 2.3–51.8%)
RTX	No	20 (54.1%)	15 (40.5%)	5 (14%; 95% CI 4.5–28.8%)
	Yes	2 (50.0%)	2 (50.0%)	0 (0%; 95% CI 0.0–60.2%)
MTX	No	19 (51.4%)	14 (37.8%)	5 (14%; 95% CI 4.5–28.8%)
	Yes	3 (75.0%)	3 (75.0%)	0 (0%; 95% CI 0.0–60.2%)

95% Cis for categorical variables and continuous variables are exact binomial (Clopper–Pearson) and Welch t-based CI, respectively SLEDAI: Systemic Lupus Erythematosus Disease Activity Index.

**Table 6:** Patient characteristics in Sjögren’s subgroup with respect to diagnostic method of TMJ disorder.

Variable		Involvement type		Difference between involvement types
		Ultrasound	Clinical	
Sex	Female	6 (66.7%)	3 (33.3%)	3 (33%; 95% CI 7.5–70.1%)
	Male	1 (100.0%)	0 (0.0%)	1 (100%; 95% CI 2.5–100.0%)
Age (years)		46.71 ± 10.42	39.00 ± 1.73	7.71 (95% CI − 1.98 to 17.40)
Disease duration (years)		5.43 ± 3.36	3.67 ± 1.15	1.76 (95% CI − 1.55 to 5.07)
Anti Ro	Negative	4 (66.7%)	2 (33.3%)	2 (33%; 95% CI 4.3–77.7%)
	Positive	3 (75.0%)	1 (25.0%)	2 (50%; 95% CI 6.8–93.2%)
Complement	Normal	6 (75.0%)	3 (37.5%)	3 (37%; 95% CI 8.5–75.5%)
	Low	1 (50.0%)	0 (0.0%)	1 (50%; 95% CI 1.3–98.7%)
RF	Negative	3 (60.0%)	1 (20.0%)	2 (40%; 95% CI 5.3–85.3%)
	Positive	1 (50.0%)	0 (0.0%)	1 (50%; 95% CI 1.3–98.7%)
CCP	Normal	4 (66.7%)	1 (16.7%)	3 (50%; 95% CI 11.8–88.2%)
	Positive	0 (0%)	0 (0.0%)	0 (0%)
Major organ	No	7 (77.8%)	3 (33.3%)	4 (44%; 95% CI 13.7–78.8%)
	Yes	0 (0.0%)	0 (0.0%)	0 (0%; 95% CI 0.0–97.5%)
PDN	No	0 (0.0%)	0 (0.0%)	0 (0%; 95% CI 0.0–97.5%)
	Yes	7 (77.8%)	3 (33.3%)	4 (44%; 95% CI 13.7–78.8%)
HCQ	No	0 (0.0%)	0 (0.0%)	0 (0%; 95% CI 0.0–97.5%)
	Yes	7 (77.8%)	3 (33.3%)	4 (44%; 95% CI 13.7–78.8%)
MMF	No	0 (0.0%)	0 (0.0%)	0 (0%; 95% CI 0.0–97.5%)
	Yes	7 (77.8%)	3 (33.3%)	4 (44%; 95% CI 13.7–78.8%)

95% CIs for categorical variables and continuous variables are exact binomial (Clopper–Pearson) and Welch t-based CI, respectively.

## 4. Discussion

Although Sjögren’s syndrome and SLE have traditionally been associated with a low incidence of erosive arthropathy, emerging evidence suggests that at least for SLE patients, up to 40% may develop erosive arthropathy over time.^[11]^ In line with this new outlook, our study revealed significant TMJ involvement in both conditions, with US detecting abnormalities at a higher rate than clinical examination, particularly Sjögren’s syndrome. Despite the limited number of cases, jaw movement restriction was observed exclusively in SLE and was absent in Sjögren’s syndrome; however, these findings should be interpreted cautiously. Given the cross-sectional design, small subgroup sizes, and the likelihood of confounding by indication and disease severity, we cannot infer medication effects on TMJ involvement. These observations are best considered hypothesis-generating and warrant confirmation in larger longitudinal cohorts with adjustment for disease activity and treatment history.

The unique structure of the TMJ, due to the fine layer of fibrocartilage overlying the articular surface, makes it susceptible to inflammatory degeneration.^[3]^ The etiological pathogenesis of TMJ dysfunction is multifactorial in systemic rheumatic diseases, with active local inflammation inducing joint space narrowing, degenerative cartilage and trabecular erosions, and condylar flattening, whereas systemic disease activity leads to myopathies that are subsequently exacerbated by chronic corticosteroid use and subsequent osteoporosis. While muscle tenderness in proximity to the joint may also signify active inflammatory processes related to TMJ dysfunction, caution is advised when attributing myofascial pain directly to TMJ involvement, given the multiple potential confounders in pain presentation.

Despite previous reports describing limited agreement between US, MRI, and clinical examination in TMJ arthritis,^[12,13]^ our findings did not replicate a lower detection rate with US compared to physical examination. Instead, US identified a higher proportion of TMJ abnormalities in both disease groups. This difference may reflect the ability of US to detect subclinical structural changes that are not apparent on routine clinical examination, particularly in early or asymptomatic disease. Although formal inter- or intra-observer reliability analysis was not performed in the present study, prior validation studies have demonstrated acceptable reproducibility of TMJ US, with reported fair-to-good interobserver reliability for the assessment of key anatomical structures^[14]^ and comparable specificity to MRI for disk pathology.^[15]^ Furthermore, a systematic review confirmed the acceptable diagnostic accuracy of US for screening disk displacement and TMJ effusion.^[16]^ In our view, a higher rate of TMJ changes on US is expected, given the asymptomatic nature of early TMJ involvement in patients. Nevertheless, MRI (particularly contrast-enhanced MRI) remains the gold standard for evaluating TMJ disorders.^[17]^

Previous studies have reported that up to two-thirds of SLE patients experience severe symptoms in relation to TM dysfunction.^[18]^ SLE was previously reported to manifest as a “stuck-jaw” sensation and restricted jaw protrusion movement.^[19]^ Nevertheless, the high heterogeneity and incompatible clinical evaluation methods in the brief review of the scarce literature on TMJ disorders in SLE by Khabadze et al and the variable prevalence of TMJ involvement in SLE in other reviews (18–85%) highlight the need for further studies.^[20]^ The correlation between the TMJ and renal involvement in SLE also provides further incentive to screen for TMJ dysfunction in these patients.^[7]^

While the limited literature generally agrees on the involvement of the TMJ in SLE,^[7]^ evidence regarding Sjögren’s syndrome remains scarce and conflicting. Historical studies failed to demonstrate a higher prevalence of clinical or radiological TMJ abnormalities compared to controls,^[21]^ and the intraoral and myofascial pain observed in later studies^[22,23]^ was often attributed to aspects of the disease other than direct TMJ involvement. However, anecdotal evidence suggests that a substantial subset of patients undergoing MRI exhibit signs of TMJ arthritis, and recent reviews indicate a higher overall prevalence of TMJ disorders (25–54%) in these patients.^[7]^ These findings align with those of our study, in which the absence of pain and tenderness over the TMJ indicates a different pattern of involvement. Our results support the direct involvement of the TMJ in Sjögren’s syndrome through an unidentified pathophysiology.

## 5. Limitations

This study has several limitations that should be considered. First, the sample size for the Sjögren’s syndrome group was much smaller compared to the SLE group. This disparity was a result of our limited temporal frame and strict exclusion criteria, specifically the exclusion of secondary Sjögren’s syndrome and patients with history of jaw trauma or dental procedures, which ensured a clean dataset but limited the statistical power for subgroup comparisons. Consequently, subgroup analyses should be interpreted as exploratory. Second, the cross-sectional design precludes us from establishing causal relationshipsbetween disease duration, medication use, and TMJ damage. Third, while we excluded active dental infections, we did not explicitly screen for or control for bruxism (teeth grinding) or malocclusion, which could act as confounders for myofascial pain. Fourth, while the radiologist was blinded to clinical data to minimize bias, inter-observer reliability was not assessed for this specific study cohort. Fifth, treatment exposure could not be evaluated as an independent correlate of TMJ findings because medication selection is strongly influenced by disease activity, organ involvement, and prior disease severity; therefore, medication-stratified comparisons are descriptive and prone to confounding by indication. Future longitudinal studies with larger, balanced cohorts are recommended to validate these findings.

## 6. Conclusion

The current study demonstrates evidence of US changes in the TMJ in more than half of the patients with SLE or Sjögren’s syndrome, a subset of which also demonstrated clinical symptoms. In view of the lack of consensus on radiological and clinical TMJ involvement in the past literature and the absence of large-scale controlled studies, the potential overlap and progression of RA in SLE and Sjögren’s syndrome, the role of DMARDs and corticosteroids in the severity of presentation, TMJ involvement in these 2 rheumatic disorders, and their association with disease activity and other findings remain an ongoing topic of investigation.

## Author contributions

**Conceptualization:** Ahmadreza Jamshidi, Tahereh Faezi.

**Data curation:** Somayeh Soroureddin, Omid Ghaemi.

**Supervision:** Majid Alikhani.

**Writing – original draft:** Ali Baradaran Bagheri.
